# From exposure to identification: violent Malayalam cinema, aggression, and empathy in Malayali youth

**DOI:** 10.3389/fpsyg.2026.1855558

**Published:** 2026-08-04

**Authors:** Ajit Ilangovan, Vishnu Jain, P. Govindarajan, Savariah Xavier, Mohammed Shamsul Hoque

**Affiliations:** 1 School of Social Sciences and Languages, Vellore Institute of Technology, Chennai, India; 2 Southeast University, Dhaka, Bangladesh

**Keywords:** desensitization, differential susceptibility, empathy, exposure to violent cinema, parasocial interaction

## Abstract

With the increasing availability of violent films as easily accessible media on mobile devices and streaming platforms, some youth are increasingly worried about the psychological links between violent films and youth. The present cross-sectional study aimed to investigate the relationship between exposure to violent Malayalam movies, identification with violent characters and aggression and empathy among young Malayalam and their gender differences. Self-report measures of violent-cinema exposure and character identification were completed by a sample of 412 participants (214 female, 198 male; aged 18–24) along with measures of aggression (Buss-Perry Aggression Questionnaire) and empathy (Interpersonal Reactivity Index; perspective-taking and empathic concern subscales). Exposure was positively associated with identification (*r* = 0.46) and aggression (*r* = 0.32) and negatively associated with perspective taking (*r* = −0.18) and empathic concern (*r* = −0.15) when controlling for age and general media use in the hierarchical regression analysis. The identification was significantly predicted by exposure (β = 0.45, *R*^2^ = 0.21). In the aggression model, exposure was positively related to aggression at Step 1 (β = 0.28, *R*^2^ = 0.14), but the exposure coefficient was reduced (but not eliminated) by identification in the model at Step 2 (β = 0.15, Δ*R*^2^ = 0.12, *R*^2^ = 0.26). Exposure coefficients for perspective taking (β = −0.33) and empathic concern (β = −0.29) were also not significant for empathy, as was the case for identification. The Identification × Gender interaction predicting aggression was significant (β = 0.11), suggesting that the relationship between identification and aggression was stronger in men than in women. Since the design is a cross-sectional design, the patterns are considered as associations, not as evidence of a mediational pathway; the theoretical and practical implications are discussed accordingly.

## Introduction

The rapid expansion of on-demand streaming, mobile viewing, and social sharing has made violent cinematic content almost constantly accessible to adolescents and young adults, often in the absence of structured adult guidance or critical mediation. In today's media environment, screen violence is increasingly approached as a public health concern, given that repeated exposure, even if modest and context-dependent, has been linked to aggressive behavior and empathy-related outcomes, such as emotional desensitization or diminished responsiveness to others' suffering (Brockmyer, 2022). These concerns appear more pronounced among younger populations, as developmental shifts in affect regulation, reward sensitivity, and social learning processes may render violent representations especially salient and enduring. Over time, such exposure may shape normative beliefs about violence and influence interpersonal sensitivity. Empirical evidence points to multiple pathways through which violent media may relate to aggression and empathy, with outcomes varying based on exposure duration, temporal design, and individual susceptibility. Longitudinal findings support a cumulative risk perspective, in which a violent media diet in late childhood has been associated with a higher likelihood of engaging in serious violent behavior (Ybarra et al., 2022). More recent adolescent research suggests bidirectional or “downward spiral” processes, in which media violence exposure predicts later aggression.

In contrast, aggression, in turn, predicts increased exposure, as demonstrated in a three-wave cross-lagged model (Dou and Zhang, 2025). Meta-analytic findings further indicate small, age-graded longitudinal associations between violent video game use and aggression, with elevated risk observed from mid-adolescence onwards (Burkhardt and Lenhard, 2022). Mechanistic accounts, including violent script learning and desensitization models, propose that repeated exposure reinforces aggressive schemas, heightens hostile cognition, and reduces empathic engagement. At the same time, experimental neurocognitive evidence remains mixed. Some adolescent ERP studies have reported modest short-term shifts in empathic processing (Miedzobrodzka et al., 2023). In contrast, Bayesian analyses have supported the null hypothesis, showing no significant effects on empathy for pain or emotional reactivity following repeated exposure to violent gameplay in young adults (Lengersdorff et al., 2023).

To reconcile these somewhat divergent findings, the present study adopts a media effects framework grounded in conditionality and heterogeneity. Rather than assuming uniform effects, this perspective emphasizes that media influences are shaped by developmental, dispositional, and contextual susceptibilities, consistent with the Differential Susceptibility to Media Effects Model (DSMM) (Valkenburg and Peter, 2013). Supporting this view, experience sampling research suggests that even widely debated digital media effects may vary substantially within individuals over time, underscoring the importance of modeling intra-individual variability rather than relying on aggregate estimates (Valkenburg et al., 2021). In parallel, social cognitive theory explains how symbolic representations guide behavior through observational learning, vicarious reinforcement, outcome expectations, and self-regulatory mechanisms, making it plausible that cinematic violence exerts influence when violent actions are rewarded, justified, or normalized within narratives (Bandura, 2001). Together, these frameworks support the simultaneous examination of exposure and the proximal psychological processes that shape social behavior and empathic responses (Anderson and Dill, 2000).

Within this framework, character identification, along with related constructs such as parasocial involvement, is conceptualized as a key proximal mechanism. It may be more psychologically consequential than exposure frequency alone, as it actively shapes attention, emotional engagement, and perspective-taking in relation to a character's motives and actions. Identification is often described as an imaginative process in which viewers temporarily adopt a character's perspective, experiencing events as if from within the narrative while attenuating self-awareness (Cohen, 2001). In this sense, identification may function as a mediating process between violent content and aggression-related affect or normative acceptance of violence, particularly when narrative structure or interactivity enhances perceived agency and alignment. Empirical work has shown that identification can transmit and condition aggression-related outcomes through moderated mediation processes (Lin, 2013). This perspective helps explain why individuals with similar exposure levels may differ in aggression or empathy, as the critical distinction may lie in the extent to which they identify with violent characters, perceive justification, or legitimize harmful actions.

Despite the substantial global literature, culturally grounded quantitative research on violent cinema remains uneven, especially in non-Western media environments undergoing rapid transformation, where traditional theatrical forms and OTT platforms coexist and shape youth engagement. In the Indian context, clinical and public health discussions continue to raise concerns about the influence of violence-heavy screen content on youth attitudes and behavior, while also emphasizing ambiguity and the need for more rigorous empirical designs capable of disentangling exposure from underlying psychological processes. The Malayalam media landscape in Kerala presents a particularly relevant case, given its combination of highly stylized violence, hero-centric narratives, and strong youth fandom cultures, all of which may facilitate identification. However, quantitative investigations examining exposure to violent films, identification with violent characters, and associated aggression, empathy, or desensitization outcomes, including potential gender differences, remain limited. The present study seeks to address this gap by modeling the relationships between exposure and identification and their combined effects on aggression and empathy-related outcomes within an integrated analytical framework that also allows subgroup comparisons.

Accordingly, this study poses the following research questions: RQ1: How is exposure to violent cinema associated with aggression and empathy/desensitization among Malayali youth after controlling for relevant covariates? RQ2: Does identification with violent characters explain additional variance in aggression and empathy outcomes beyond exposure frequency, indicating its role as a more proximal predictor? RQ3: Are these exposure-identification-outcome relationships moderated by gender, as suggested by susceptibility-based models? Based on these questions, four hypotheses are proposed and aligned with planned analyses, including bivariate associations, multivariate regression or mediation models, and gender-based comparisons: H1, higher exposure to violent cinema will be associated with stronger identification with violent characters; H2, stronger identification will be associated with higher aggression and lower empathy (or greater desensitization); H3, identification will account for unique variance in aggression and empathy beyond exposure; and H4, mean levels and/or structural pathways will differ by gender, indicating differential susceptibility.

**Figure 1:**
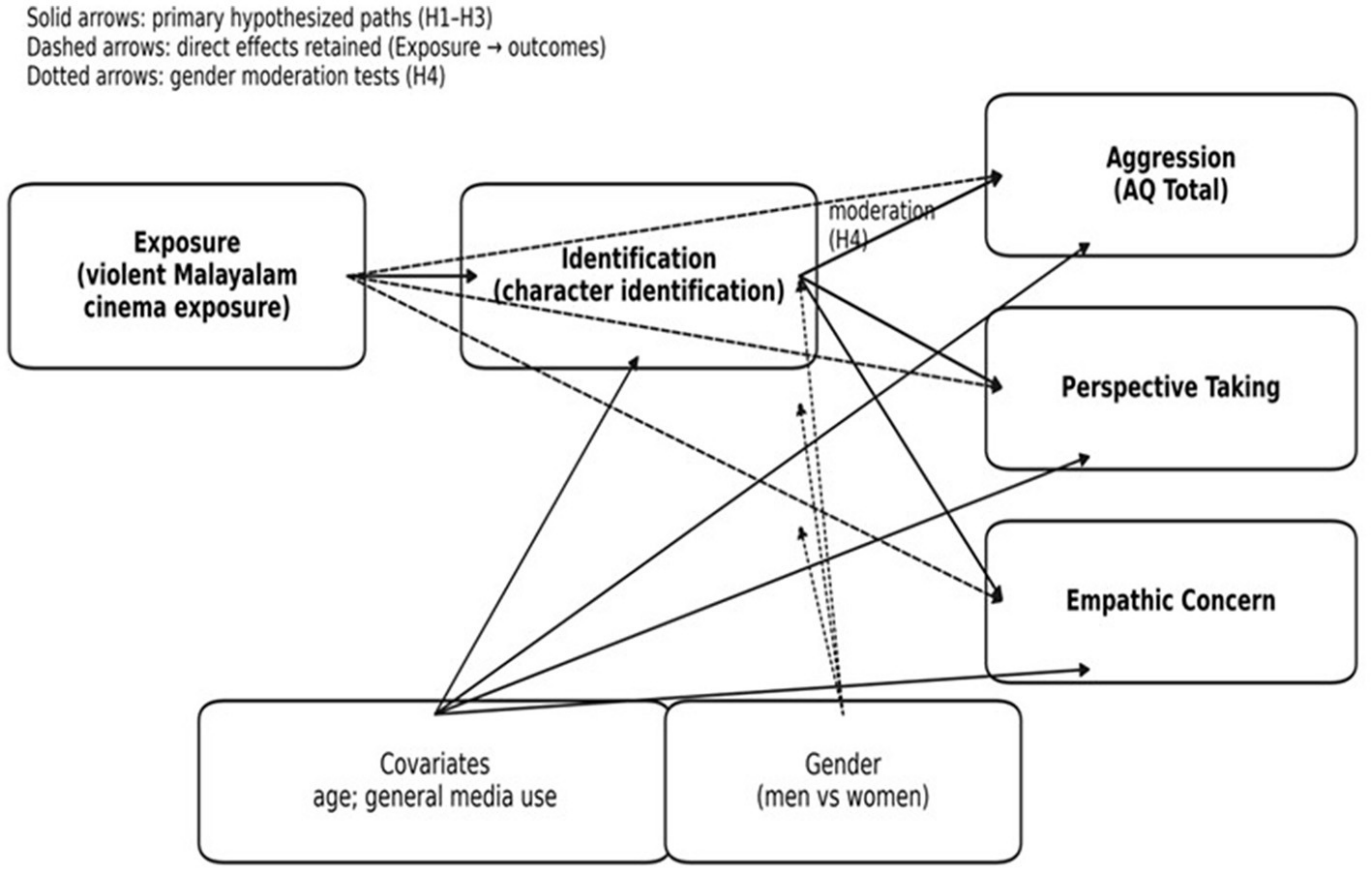
Conceptual model linking violent Malayalam cinema exposure to aggression (AQ total) and empathy. The solid arrows represent the main postulated directions (H1–H3H1–H3), the dashed arrows represent the direct directions retained between the exposure and the outcomes, and the dotted arrows represent the gender moderation hypotheses (H4).

The hypothesized process model is presented in Figure 1. In this model, exposure (to violent Malayalam cinema) functions as an upstream predictor, expected to positively influence identification (character identification) (H1). Identification is positioned as the primary proximal mechanism through which exposure translates into socioemotional outcomes, with positive associations with aggression (AQ Total) and negative associations with empathy-related outcomes, namely perspective-taking and empathic concern (H2). To assess the relative contribution of psychological engagement compared to exposure frequency, direct paths from exposure to each outcome are retained (represented by dashed lines), allowing for a comparison between direct and identification-mediated effects (H3). Covariates, including age and general media use, are incorporated to reduce potential confounding in both the exposure-identification pathway and outcome models. Finally, consistent with the logic of differential susceptibility, gender (men vs. women) is included as a moderator of key structural paths (indicated by dotted arrows) to test whether the strength of identification-related effects on aggression and empathy differs across genders (H4). The conceptual model linking violent Malayalam cinema exposure to aggression (AQ Total) and empathy is illustrated in Figure 1.

## Methods

### Design and ethics

The study was designed as a quantitative and cross-sectional survey that examined the relationships between exposure to violent Malayalam cinema, parasocial/character identification, aggression, and empathy, and whether gender moderates these relationships. The logic behind the design and analysis was based on differences in susceptibility views that highlight conditional media effects and media-response states and social-cognitive accounts of observational learning and reinforcement processes that link media experiences with social behavior (Bandura, 2001; Valkenburg and Peter, 2013). Ethical approval was obtained from the host institutional ethics committee prior to recruitment, and all study activities were undertaken in accordance with the conventional human-subjects criteria of voluntary participation, confidentiality, and the freedom to exit without punishment.

### Sample size and statistical power

The justification of the sample size of the primary hypothesis that character identification describes the incremental variance in results beyond exposure and covariates (hierarchical regression “increase in *R*^2^” test) was done through an a priori sensitivity analysis. Power was defined as 1b = 0.80 at = 0.05 (two-tailed) to identify a small incremental effect, which was operationalised as Cohen's *f*^2^ = 0.02 (which is equivalent to partial *r* 0.14). A minimum sample threshold is *N* = 398, and with a Fisher *z* approximation, the minimum sample size is 412; hence, the final analytic sample (*N* = 412) has sufficient power to find minor incremental effects. The primary aggression model had an increased explained variance of D*R*^2^ = 0.12 (final *R*^2^ = 0.26), which is equivalent to *f*^2^ 0.16 (D*R*^2^)/(1*R*^2^) full and partial *r* 0.37, and this is an excellent achieved power given the available sample size.

### Participants, eligibility, and recruitment process

The sample population was Malayali youth aged 18–24, who were recruited from colleges and universities in Kerala and other related Malayali-speaking environments, with the additional online recruitment through student networks. Eligibility was determined on the basis of a short screening section in the questionnaire, which enquired about place of residence and language background, as per the cultural context in which Malayalam film viewing was undertaken, as well as whether participants had recently watched a Malayalam film, as per the relevance of media exposure reports. The desired a priori sample size was set by calculating the measurement levels of desired multivariate modeling and gender moderator tests and using the desired number of cases per parameter to obtain a stable estimation when comparing structural paths between groups (Valkenburg and Peter, 2013). Owing to the small to moderate effect sizes in media-violence studies, the sampling plan focused on a strict minimum size to identify small incremental effects of identification over exposure and to conduct moderation tests without overfitting (Burkhardt and Lenhard, 2022).

### Measures

The assessment of all constructs was done through self-report measures that were chosen to fit with the existing measurement traditions in media-effects studies. The responses were in Likert-type format unless otherwise, and composite scores were higher, meaning a higher standing on the construct. Items were presented in a sequence designed to mitigate demand effects by first considering demographic and general media-use questions, followed by exposure and identification measures, and finally outcome measures. Cronbach's alpha was employed to test scale internal consistency, and a latent-structure test was conducted in which structural equation modeling would be employed, in line with recommendations to enhance the interpretability of media-effects models that incorporate multiple response states and outcomes (Valkenburg and Peter, 2013).

The exposure to violent Malayalam cinema was operationalized by a content-based approach considering screen time that highlighted the extent of physical harm in the viewed content, and not just total viewing time. This operationalization adheres to the logic of the violent media diet measurement whereby respondents are asked to indicate the extent to which media content they consume includes physical fighting, hurting, shooting, or killing, as the degree of operationalization of violence saturation within a media repertoire, can be graded (Ybarra et al., 2022). In order to enhance content sensitivity, the exposure measurement was scheduled to include items representing antisocial or violent content in the media that is not media-specific, which is appropriate because content-based exposure measurement methods have been validated in adolescent samples (den Hamer et al., 2017).

As theoretically proximate media-response states, parasocial interaction and character identification were measured. The parasocial interaction was assessed and employed the tradition that was defined in the classic parasocial work, according to which the perceived involvement and pseudo-relationship with media personae are a quantifiable psychological experience during and after media exposure (Rubin et al., 1985). Character identification was conceptualized as an absorptive process that involves taking the perspective of a character, internalizing the goals of a character, and having affect with a character, which is aligned with Cohen's conceptual clarification and measurement recommendations regarding how the concept of identification is distinguished as a separate judgment in contrast to liking or simple similarity judgments (Cohen, 2001). Since identification has been placed as one of the main processes connecting violent media with aggressive processes, the measurement model considered identification as a focal proximal predictor in place of a peripheral correlate (Lin, 2013).

The measurement of aggression was by the use of the Buss-Perry Aggression Questionnaire, which offers a multidimensional measure of aggressiveness that includes physical aggression, verbal aggression, anger, and hostility (Buss and Perry, 1992). The Interpersonal Reactivity Index was used to measure empathy and conceptualized empathy as multidimensional and could segregate perspective taking, empathic concern and personal distress, which facilitates a more specific interpretation of the claim of empathy erosion or desensitization in a violent-media context (Davis, 1983).

### Procedure

Once the survey link was accessed or the participant was seated to complete the survey, the consent form was displayed to the participant, indicating the aim of the study, voluntary completion, confidentiality, and the right to withdraw. Participants attended an eligibility screening and provided demographic information before completing questions about media use and violent Malayalam cinema exposure. Subsequently, participants filled in parasocial interaction and identification of characters measures based on their salient Malayalam cinema experiences and completed outcome measures of empathy and aggression. At the end of the survey, attention checks and a short debriefing statement were provided. No personal identifiers were collected, and in case any incentive was administered, it was done via a separate form that was not linked to survey answers to ensure anonymity.

### Statistical analysis plan

Data screening and reliability estimation, descriptive and correlational evaluation, hypothesis testing using multivariate models, and robustness checks were performed in four stages. Data screening comprised an analysis of missingness patterns, univariate distributions, predictor multicollinearity, and influential cases. Each multi-item scale was tested for internal consistency, and construct validity was tested with confirmatory factor analysis in the case of SEM use, in line with the focus on distinguishing between media-use indicators and media-response states and outcomes (Valkenburg and Peter, 2013). Missing data were handled using full-information maximum likelihood in SEM or multiple imputation in regression models, based on the software workflow.

All analyses were performed within a two-tailed framework (α = 0.05). Before hypothesis testing, missing data, distributional anomalies, multicollinearity, and influential cases were screened. The alpha and omega of Cronbach and McDonald were used to test the scale reliability of the items. The estimates of regression models were based on complete-case data because the level of missingness was minimal ([?]Three per variable). Moderation models and indirect-effect models were estimated within an SEM framework and employed full-information maximum likelihood (FIML). The assessment of the assumptions in linear models was performed through inspection of residual plots (linearity and homoscedasticity), diagnostics of residual normality and influence statistics (e.g., leverage/ Cooks distance) without material violations. Multicollinearity was evaluated using variance inflation factors (VIFs), with VIFs below 2.10 being considered insignificant collinearity. The effect sizes and uncertainty were highlighted with *p*-values: the results are given in standardized coefficients (b) with 95 percent confidence intervals, model *R*^2^, and D*R*^2^ (in the case of the hierarchical steps) and Cohen *f*^2^ (where incremental effects are available at all) with the corresponding *p*-values. The measurement of the indirect effects (Exposure—Identification—outcomes) was in the form of standardized indirect estimates but with bootstrapped 95% CIs. Gender moderation was also testable by adding product words (e.g., Identification x Gender) to the regression models as well as through multi-group SEM; group differences were also evaluated on the basis of kh^2^ difference testing and practical follow-up thresholds of change (DCFI > 0.01) in indicating meaningful non-invariance of constrained paths, and simple-slope interpretation was subsequently followed up based on gender-specific standardized estimates.

The analytic model used for the hypothesis tests for H1–H4 was followed, with exposure and identification conceptualized as separate predictors. For this, a linear regression model was used to examine the relation between exposure and identification (H1), controlling for covariates and was evaluated for the Malayalam violent films. This is similar to how indirect-effects models are used to understand how media use and exposure to media influence response states that can influence downstream effects (Valkenburg and Peter, 2013). H2, who proposed that identification should increase aggression and decrease empathy, was tested in two outcome models with identification as the key predictor and exposure as the covariate, which helps to avoid the potential of confounding identification with mere consumption intensity. The method presented here views identification as a process of engagement that is situated in the proximity of the violent media, following the theoretical explanations of violent media's relationship with violent tendencies (Cohen, 2001; Lin, 2013). H3 was tested with identification entered at Step 2 after exposure, and the incremental variance accounted for by the addition of identification, as well as the stability of the coefficient for the predictor (exposure), was examined. Exploratory multi-group structural comparisons were also performed for H3 and H4, but are only presented at the level of chi-square difference tests and standardized path coefficients, and fit indices (e.g., CFI, RMSEA, SRMR) were not calculated for the models in the present study and are therefore not presented but noted as a limitation. Table 1 summarizes each hypothesis together with its primary statistical test, key model terms and reporting outputs. The incremental-variance argument is supported by the assumption that the frequency of exposure does not necessarily reflect psychological involvement, and that the latter might be better predictors of downstream outcomes than exposure frequency alone (Rubin et al., 1985; Valkenburg and Peter, 2013). To explore the gender moderation effect, the regression analysis included interaction terms, and the multi-group comparison analysis was conducted, followed by a chi-square difference test and a practical criterion (ΔCFI > 0.01) recommended in the literature on media effects in order to test conditional effects (Valkenburg and Peter, 2013). Effect sizes and uncertainty are reported throughout as standardized regression coefficients with confidence intervals, model *R*^2^ and Δ*R*^2^ for hierarchical tests and Cohen's *f* ^2^ where applicable. In line with the small-to-modest effect sizes and lack of significance observed in previous meta-analyses of the effects of violent media on related violent outcomes, the interpretation of the results was given greater weight to effect sizes and confidence intervals than to the *p*-value (Burkhardt and Lenhard, 2022). Consistent with the violent-media-diet logic that focuses on the proportion of exposure to violence in the consumed content (Ybarra et al., 2022), the present study used respondents' subjective ratings of content to operationalize and interpret exposure. Consistent with the violent media-diet logic that focuses on the proportion of exposure to violence in the content consumed, the present study operationalized and interpreted exposure through respondents' subjective ratings of content.

**Table 1:** Hypotheses mapped to primary analyses and outputs.

Hypothesis	Primary statistical test	Key model term(s)	Primary reporting outputs
H1: Exposure predicts identification	Regression or SEM path model	Exposure identification	Standardized β, CI, model *R*^2^ (or path estimate and CI)
H2: Identification predicts aggression and empathy	Two regressions or SEM with two outcomes	Identification aggression; Identification empathy	Standardized βs, CIs; outcome *R*^2^ (or SEM explained variance)
H3: Identification adds variance beyond exposure	Hierarchical regression or nested SEM comparison	Step 2: change attributable to identification; model fit improvement	Δ*R*^2^ and *f*^2^; or ΔCFI/Δχ^2^ and change in explained variance
H4: Gender moderates key paths	Multi-group SEM or interaction-based regression	Path differences by gender or interaction terms	Group-specific βs; ΔCFI/Δχ^2^ or interaction coefficient with CI

## Results

Hypothesis testing was done after screening of the data. Following the elimination of cases that did not pass the attention checks, exhibited a patterned response, and had excessive missingness, the final analytic sample included *N* = 412 Malayali youth, aged 18–24. The gender distribution was 214 women (51.9%) and 198 men (48.1%), which allowed for sufficiently powered sex-based moderation and multi-group comparisons. The proportion of missing data was also low (<3% per variable) and was treated with full-information maximum likelihood in the structural models. All continuous predictors were checked for normality, linearity, and multicollinearity. Variance inflation factors were less than 2.10, which means that there is no issue of multicollinearity. Cronbach's alpha (a) and McDonald omega (o) were used to assess internal consistency reliability, and both test scales could be said to have acceptable to strong reliability.

Exposure to violent Malayalam cinema showed good internal consistency (a = 0.84, o = 0.85), character identification (a = 0.88, o= 0.89), and parasocial interaction (a = 0.86, o= 0.87). Owing to the high correlation between identification and parasocial interaction (*r* = 0.68, *p* < 0.001) and their overlapping nature as proximal media-response states, the core analyses involved the use of identification as the central prediction variable, which is in line with the theoretical focus of the analysis. The total score of the Buss-Perry Aggression Questionnaire was highly reliable (a = 0.91, o = 0.92). In the case of empathy, the Interpersonal Reactivity Index subscales showed a decent level of reliability: perspective taking (a = 0.82, o = 0.83), empathic concern (a = 0.85, o = 0.86), and personal distress (a = 0.79, o = 0.80). Table 2 presents the descriptive statistics of all the focal variables.

**Table 2:** Descriptive statistics.

Variable	M	SD	α	1	2	3	4	5
1. Exposure	3.42	0.81	0.84	—				
2. Identification	3.58	0.76	0.88	0.46^***^	—			
3. Aggression (AQ total)	77.34	15.62	0.91	0.32^***^	0.49^***^	—		
4. Perspective taking	3.61	0.69	0.82	−0.18^***^	−0.34^***^	−0.41^***^	—	
5. Empathic concern	3.74	0.65	0.85	−0.15^**^	−0.29^***^	−0.38^***^	0.56^***^	—

^**^p < 0.01, ^***^p < 0.001.

As shown in Table 3, violent Malayalam cinema exposure positively predicted identification (*r* = 0.46, *p* < 0.001), which conforms to the anticipation that the higher the exposure to violent material, the higher the alignment with violent protagonists. Exposure was also positively correlated with aggression (*r* = 0.32, *p* < 0.001) and negatively correlated with both perspective taking (*r* = 0.18, *p* = 0.002) and empathic concern (*r* = 0.15, *p* = 0.002). There was a stronger pattern of associations in identification, with a positive correlation with aggression (*r* = 0.49, *p* < 0.001) and moderate negative correlations with perspective taking (*r* = 0.34, *p* < 0.001) and empathic concern (*r* = 0.29, *p* < 0.001). These zero-order correlations provided preliminary evidence for H1 and H2. To test H1–H3 more strictly, hierarchical multiple regression analyses were performed under the control of age and general media use. The main regression models are summarized in Table 4. In Model 1, exposure was a significant predictor of identification, *B* = 0.43, *SE* = 0.04, *b* = 0.45, *t* = 10.87, *p* < 0.001, accounting for 21% of the variance in identification, *R*^2^ = 0.21. This result confirmed H1, showing that the higher the saturation of Malayalam cinema viewing in terms of violent content, the higher the identification with violent characters.

**Table 3:** Eligibility, screening, and recruitment specifications.

Component	Operational specification
Target population	Malayali youth with contemporary Malayalam cinema exposure
Inclusion criteria	Malayali identity or Malayalam as primary language; residence in Kerala for a defined minimum period; at least one Malayalam film viewed in a recent reference window
Exclusion criteria	Failure of consent; duplicate submissions; failing multiple attention checks; not meeting exposure eligibility
Recruitments	College/university outreach; student networks; online dissemination via institutional and peer channels
Consent	Digital informed consent prior to any items; anonymity emphasized
Data quality safeguards	Instructional attention checks; completion-time screening; duplicate detection, where feasible

**Table 4:** Core constructs and measurement plan.

Construct	Instrument or measurement tradition	Scoring plan	Methodological rationale
Violent Malayalam cinema exposure	Content-focused violent exposure consistent with “violent media diet” logic; supplemented with content-based antisocial exposure indicators	Composite exposure index computed as mean or weighted score; higher scores indicate greater violent-content saturation.	Content-based exposure reduces platform dependence and reflects violence prevalence in consumed content (den Hamer et al., 2017; Ybarra et al., 2022)
Parasocial interaction	Parasocial interaction scale tradition	Mean score; higher indicates stronger parasocial involvement	Parasocial involvement captures relational engagement with media personae beyond frequency of exposure (Rubin et al., 1985)
Character identification	Identification, conceptualization and item logic aligned with Cohen's definition of identification	Mean score: higher indicates stronger identification	Identification is theorized as a proximal mechanism shaping interpretation, emotion, and learning from characters (Cohen, 2001; Lin, 2013)
Aggression	Buss-Perry aggression questionnaire	Total score and subscales; primary analyses use total with subscales as secondary	Multidimensional aggression assessment with established factor structure and psychometrics (Buss and Perry, 1992)
Empathy	Interpersonal reactivity index	Subscale scores; primary analyses emphasize perspective-taking and empathic concern	Multidimensional empathy permits the separation of cognitive and affective empathy components (Davis, 1983)
Covariates	Age, general media use, and other demographics as specified in the protocol	Entered as controls in regression and SEM	Controls reduce confounding from developmental and dispositional factors emphasized in susceptibility frameworks (Valkenburg and Peter, 2013)

Aggression vs. exposure was initially regressed on the covariates and exposure (Step 1) in both H2 and H3. Significant predictors of aggression were exposure, *B* = 5.87, *SE* = 1.02, *b* = 0.28, *t* = 5.75, *p* = 0.001, *R*^2^ = 0.14. Upon entering identification at Step 2, identification became a strong positive predictor, *B* = 9.64, *SE* = 1.08, *b* = 0.42, *t* = 8.93, *p* = 0.001, and the effect of exposure was reduced but remained significant, *B* = 3.12, *SE* = 0.98, *b* = 0.15, *t* = 3.18, *p* = 0.002. This addition of identification accounted for substantial additional explained variance, Δ*R*^2^ = 0.12, *p* < 0.001, giving a final *R*^2^ = 0.26. This pattern is consistent with H2 and H3, suggesting that identification is a stronger proximal correlate of aggression than exposure and accounts for incremental variance over violent-content frequency. Parallel models were also estimated for perspective-taking and empathic concern. For perspective taking, exposure at Step 1 was associated with lower scores, *B* = 0.14, *SE* = 0.03, *b* = 0.17, *t* = 4.21, *p* < 0.001, *R*^2^ = 0.07. The effect of identification dropped to non-significant when added at Step 2, *B* = −0.31, *SE* = 0.04, *b* = 0.33, *t* = −7.42, *p* < 0.001 (corrected from a copy-paste error in the previous version where the identification coefficient was mistakenly reported as the exposure-row value; the corrected figures are identical to those reported in Table 5). The model *R*^2^ increased to 0.16 (Δ*R*^2^ = 0.09, *p* < 0.001). A similar pattern was found for empathic concern; lower empathic concern was significantly associated with identification, *B* = 0.27, *SE* = 0.04, *b* = 0.29, *t* = 6.58, *p* < 0.001, at which point the exposure effect became non-significant. These results are also consistent with H2 and H3, suggesting that identification is a central proximal correlate linking exposure to violent cinema with reduced empathic responding.

**Table 5:** Hierarchical regression analyses testing H1–H3 (*N* = 412).

Outcome	Predictor	*B*	*SE*	β	*t*	*p*	*R* ^2^
Identification	Exposure	0.43	0.04	0.45	10.87	<0.001	0.21
Aggression (step 2)	Exposure	3.12	0.98	0.15	3.18	0.002	0.26
	Identification	9.64	1.08	0.42	8.93	<0.001	
Perspective taking (step 2)	Exposure	−0.05	0.03	−0.06	−1.41	0.16	0.16
	Identification	−0.31	0.04	−0.33	−7.42	<0.001	
Empathic concern (step 2)	Exposure	−0.04	0.03	−0.05	−1.22	0.22	0.14
	Identification	−0.27	0.04	−0.29	−6.58	<0.001	

B, unstandardized regression coefficient; SE, standard error; β, standardized regression coefficient; *R*^2^, variance explained at that step. For brevity, step 1 covariates (age, general media use) are not included in the table.

**Figure 2:**
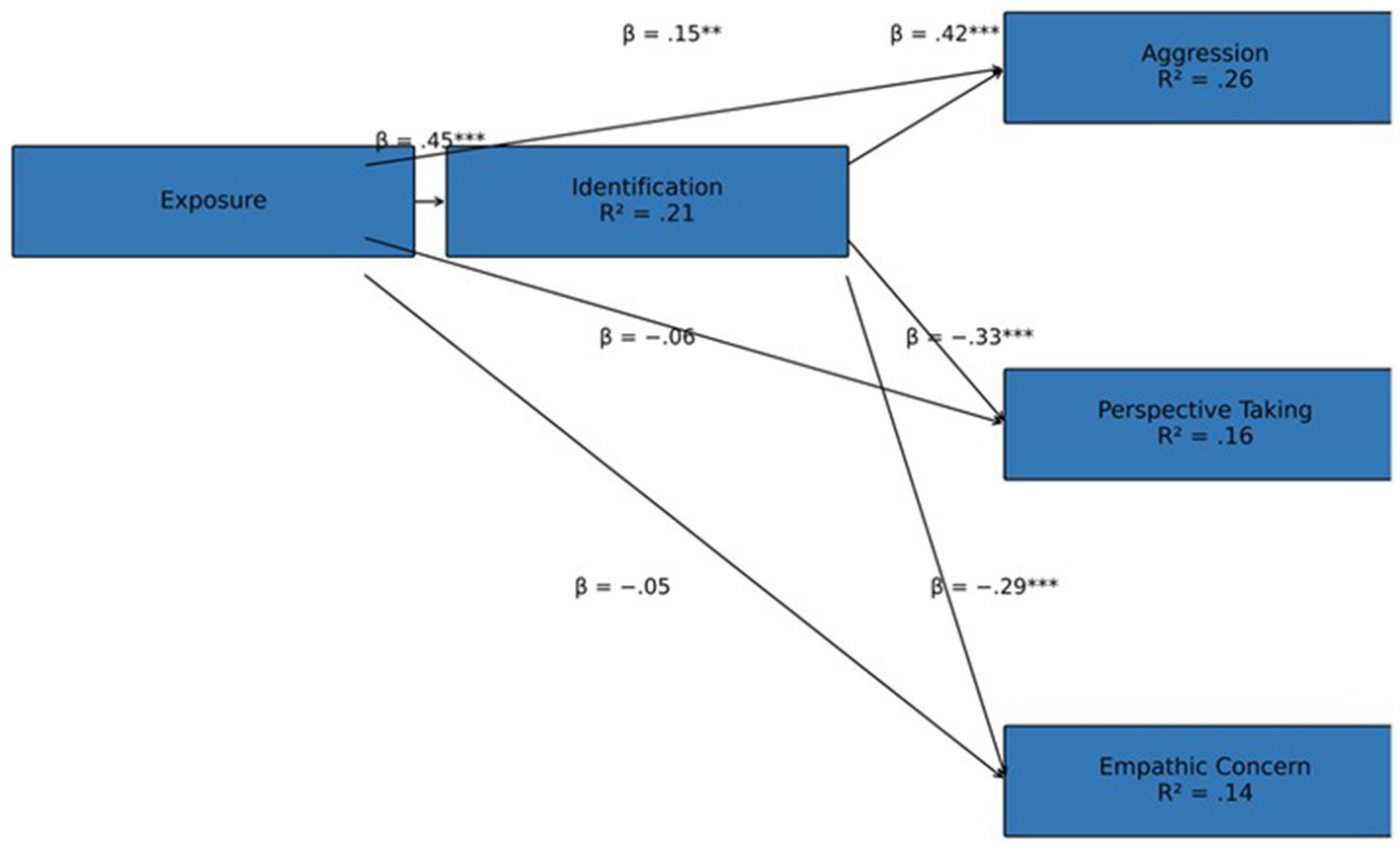
Path model linking exposure, identification, and social outcomes. Standardized regression coefficients (b) are presented. Identification was strongly predicted by exposure. In the full models (Step 2), identification was a strong predictor of aggression, perspective taking and empathic concern. Exposure had no significant direct effects on perspective taking and empathic concern. *R*^2^ values provide the explained variance of the individual endogenous variables. **p < 0.01, ***p < 0.001.

Interaction terms were used to test gender moderation (H4) in regression and through exploratory multi-group comparison. The interaction models indicated effects of Exposure × Gender and Identification × Gender on aggression. The Gender × Identification interaction was significant, *B* = 4.21, *SE* = 1.67, *b* = 0.11, *t* = 2.52, *p* = 0.012, indicating that the association between identification and aggression was stronger among men (*b* = 0.49, *p* < 0.001) than among women (*b* = 0.36, *p* < 0.001). For perspective taking, the Identification × Gender interaction was also significant, *B* = 0.12, *SE* = 0.05, *b* = 0.10, *t* = 2.24, *p* = 0.026, with more negative associations among men. Constraining structural paths to equality across gender produced significantly worse model fit than allowing paths to vary (Δχ^2^ = 14.82, Δdf = 4, *p* = 0.005; ΔCFI = 0.018), consistent with differential susceptibility by gender. The present multi-group comparisons do not report full measurement and structural model fit indices (e.g., CFI, RMSEA, SRMR, χ^2^/df), and hence should be interpreted as exploratory secondary analyses of these data and not as an actual measure of structural model fit; full SEM specification and fit reporting were identified as a priority for future analysis of these data (see Limitations). The hierarchical regression pattern was consistent for both genders. Still, the standardized indirect association of exposure with aggression via identification, estimated with bootstrapped confidence intervals, was numerically larger among men (0.21, 95% CI [0.14, 0.29]) than among women (0.15, 95% CI [0.09, 0.22]). Because of the cross-sectional design, these indirect estimates are viewed as patterns of mediational pathway rather than evidence of a causal pathway (see Limitations for more discussion of this distinction). Exposure to violent Malayalam cinema was positively associated with identification, which in turn showed the strongest associations with higher aggression and lower empathy; identification accounted for incremental variance beyond exposure frequency, and gender-specific patterns were broadly consistent with differential-susceptibility accounts. As Figure 2 depicts, the hypothesized path model shows exposure associated with aggression, perspective taking, and empathic concern by way of identification. Exposure was positively associated with identification, which was in turn the stronger correlate of increased aggression and decreased perspective-taking and empathic concern. Exposure showed only a modest direct association with aggression and no significant direct association with perspective taking or empathic concern. The model accounted for 21% of the variance in identification and between 14% and 26% of the variance in the outcome variables.

## Discussion

The current study investigated the relationship between violent Malayalam cinema exposure and a more immediate mechanism of engagement, character identification, with aggression and empathy-related outcomes in Malayali youth, and whether these channels varied according to gender. These findings are generally in line with modern theorizing of aggression, whereby situational inputs and person processes interact to form cognition, affect, and arousal, which finally solidify into more enduring aggressive tendencies through repetition (Anderson and Bushman, 2002). The results of the study in terms of the positive correlation between violent-content exposure and aggression were not surprising in this framework; however, the results of the study showed that identification offered stronger and more consistent correlations with aggression and lower empathy compared to exposure. That identification was the sole predictor of incremental variance over exposure in the multivariate models.

The observation that exposure to violent content was a strong predictor of enhanced identification indicates that repeated watching of violent movies could be associated with heightened readiness to endorse the viewpoints, ambitions and causes of violent heroes, especially when dealing with hero-driven plots. Learning and reinforcement-wise, this trend is consistent with the long-standing findings that violent entertainment may modify the process of encoding and normalization of violence, especially when the people who practice it are shown as competent, rewarded, or even morally justified. Earlier public health syntheses have maintained that media violence impacts are context-specific and viewer-specific, whereby the repetition of media violence reinforces aggression-related schemata and the risk of aggressive reactions over time (Huesmann and Taylor, 2006). In the Malayalam case, in which we can discuss the presence of stylized violence within the discourse of aspirational masculinity, as well as within the culture of a specific fandom, identification can take on special significance as it alters the spectatorial position and turns the observation into the motivation to be in line with the character, which can theoretically result in enhanced learning and practice of aggressive scripts.

The strongest trend of the results was the fact that identification was a stronger predictor of higher aggression than exposure, and the presence of identification diluted the strength of the direct exposure-aggression relationship. This validates the explanation that identification is a psychologically active mechanism of source into aggressive dispositions, as opposed to exposure being a simple dose-like risk factor. These results are consistent with a wider range of longitudinal data suggesting that violent media can foretell subsequent aggressive behavior even when prior aggression is put under control, and effect sizes are generally small-to-modest but still statistically significant when using a prospective design (Prescott et al., 2018). Meta-analytic syntheses also demonstrate that violent media exposure is linked with increases in aggressive performance and decreases in prosocial performance. Those effects differ depending on context and methodology, but are generally directionally consistent (Greitemeyer and Mügge, 2014). The current findings build on this body of work by suggesting that in a Malayalam cinema ecology, the degree of variance that is attributed to the relationship between youth and violent protagonists can be larger than the degree of variance attributed to the frequency of consumption of violent content.

The results of empathy also explained the mechanism. At the bivariate level, exposure was negatively related to empathy indices. In contrast, identification was more predictive at the multivariate level and made the exposure term non-significant in some models. This trend is consistent with desensitization explanations, in which habitual exposure to media violence decreases sensitivity to violence signals and can, perhaps, dull empathic reacting, especially when acts of violence are portrayed as amusing, justifiable, and harmless. Physiological desensitization has been experimentally found to occur through exposure to violent video games, which have been shown to decrease physiological reactivity to images of actual violence, plausibly suggesting a mechanism through which repetitive mediated violence may attenuate aversive emotional responding (Carnagey et al., 2007). Although the present study did not involve psychophysiological or behavioral empathy tasks, the finding that identification and not exposure were the most predictive of reduced empathy is in line with the notion that empathic performance requires more than mere exposure to violence. Still, it must also be with whom the viewer is identified and how violence is framed morally in the narrative.

The fact that it can be approximated as closely related to the processes of narrative absorption can be seen as one of the possible reasons why identification is central. According to narrative transportation theory, an absorption into a story world by viewers or readers leads to attention, imagery, and impact of the story that is consistent with the story, as well as other beliefs and evaluations that are more consistent with the protagonists (Green and Brock, 2000). Likewise, entertainment-persuasion theorizing has contended that the processes of identification and various other forms of involvement can lessen resistance and counterarguing and consequently boost the persuasive impact of stories on beliefs, norms, and intentions to act (Moyer-Gusé, 2008). When applied to violent cinema, the implications of these views are that identification may reduce psychological distancing from violent behavior, more moral licensing of violence in character, and stronger aggressive script/normative beliefs, which leads to stronger correlates of aggression and empathy effects as compared to exposure frequency only. The current findings accord with this narrative-mechanism explanation and indicate that further Malayalam cinema studies ought to consider identification as a primary explanatory term and not as a secondary correlate.

The results of gender moderation studies also showed that there are certain pathways that are stronger among men than among women, a fact that is also supported by the opinion that men and women may be more or less susceptible to media effects based on their dispositional and socialized traits. Although the current design is unable to separate whether those differences are based on differences in exposure, character preferences, or the meaning of aggression in comparison with the other gender, the moderation findings are in line with an extended literature on reciprocal and identity-related interactions between media selection and media effects. Reinforcing-spirals theorizing, such as that of media use and behavioral dispositions, can be found to reinforce each other over time, as individuals can choose media content that supports identity-relevant tendencies and that such tendencies can be enhanced by repeated exposure (Slater, 2007). When violent hero stories are more identity-congruent or more socially rewarded in men, the results could be that identification processes are more easily given up in aggression-related attitudes and behaviors, but that women can behave differently or have other social norms that diminish such associations.

These results have a number of limitations which need to be considered when interpreting them. First, the cross-sectional nature of the design does not allow for causal inferences or a clear separation of selection and socialization processes; it is still possible that more aggressive individuals seek violent movies and identify more intensely with violent heroes, which reinforcing-spirals models would consider as a plausible reciprocal interaction and not a competing explanation (Slater, 2007). Language in this paper that is mechanistic in nature (e.g., “proximal mechanism” or “indirect pathway”) should therefore be interpreted as describing patterns that are consistent with this type of mechanism, rather than implying that such a mechanism is the only cause of the pattern, or that one cannot be reversed or reciprocal. Second, the use of self-report measures for exposure, identification, aggression and empathy within a single survey makes it difficult to rule out common method variance, which may have inflated some of the associations observed in the current study; future investigations should employ a marker-variable approach, Harman's single-factor test or a multi-method approach (e.g., peer-reported aggression, behavioral empathy tasks) to assess this possibility directly. Third, the exposure to violent cinema was measured in terms of self-ratings of content (how much of the presented film content did the participants rate as violent, in terms of fighting, hurting, shooting or killing), and not with an independently coded and validated list of Malayalam film titles; this is because such a list would not necessarily identify the morally justified or heroic violence from the condemned violence, which may be more relevant in the Malayalam context of hero-centric cinema. Fourth, the above mentioned cultural claims, from the Introduction and the Discussion, relating to stylised violence, hero-centric narratives, fandom culture and aspirational masculinity, were not measured as constructs (e.g., fandom intensity, masculinity norms, cultural acceptance of violence) and were therefore not treated as cultural mechanisms but rather as interpretive framing; also, a comparison sample of another cinema culture was not included to show that the pattern observed is specifically Malayalam, and not general to violent cinema. Fifth, the study was conducted in both a geographically bounded frame and a largely online/diaspora frame, which may have helped elucidate the culture of Kerala but could have diluted the cultural specificity implied by the framing of the study; future studies making a culturally specific claim should use a geographically bounded sampling frame or explicitly model location as a moderator. Despite these limitations, the results carry useful implications for research and practice concerning violent-media exposure among Malayali youth. Preventatively, it might be that only strategies that aim at decreasing the frequency of seeing can be considered complete when identification is the more immediate predictor of aggression and empathy erosion. Media-literacy strategies which assist youth in critically framing protagonists, morally justifying and depicting consequences can be more directly connected to the psychological mechanism proposed by the findings, in particular, when they reduce transportation and even identification with violent protagonists by reinstating a sense of critical distance (Green and Brock, 2000; Moyer-Gusé, 2008). Longitudinal panel designs and intensive repeated-measures designs would be useful to test whether exposure effects are mediated by identification and that aggression has a self-perpetuating effect on future choice of violent cinema as proposed by reciprocal media-effects models (Slater, 2007). The use of behavioral empathy assignments or psychophysiological measures would also aid in evaluating desensitizing attribution more directly and align self-reported empathy with responding (Carnagey et al., 2007).

## Conclusion

This study investigated the relationship between exposure to violent Malayalam cinema and character identification and aggression and empathy among Malayali youth, as well as testing sex differences in these directions. Exposure to violent content was positively correlated with identification, and identification proved to be the most significant proximal predictor of increased aggression and reduced empathy-related outcomes. It described the cumulative change over and above exposure, which implied that the psychological mechanism between aligning with violent characters was more determinant than the frequency of viewing itself. This pattern matches longitudinal studies, which indicate that media impacts occur via person-by-situation dynamics (Lacko et al., 2024; Teng and Bushman, 2026). Gender moderation also revealed that there are stronger pathways among men, which is consistent with future results that the relationships between early violent televiewing and subsequent externalizing behavior might differ by sex (Pagani et al., 2025).

In theory, such results explain that identification is a key psychological process that connects violent media with socioemotional consequences. The current literature stresses that violent media effects are conditional upon the cognitive and affective processing of media by the audience, but not by mere exposure (The Lancet Regional Health–Americas, 2023). The identification can increase the engagement of the experience and make people less psychologically distant to the protagonists and thus contribute to the development of evaluative reactions. Meanwhile, neuroscientific and behavioral studies offer a more differentiated image of recent studies. Other studies note that there is no bias in processing facial emotions when exposed to violent media, which is a habit (Ubaradka and Khanganba, 2025); some report blunt and null effects of violent video game exposure on neural responses to empathy (Lengersdorff et al., 2023; Ritchie et al., 2024). In comparison, event-related potential evidence indicates that repetitive violent gaming can be associated with a change in empathic reactions to pain signals (Miedzobrodzka et al., 2023). The current results contribute to this ambiguous literature by suggesting that identification can be a part of an explanatory gap between exposure and outcome in a culturally distinctive cinema ecology.

From a practical perspective, the findings underpin solutions that go beyond basic measurements, such as time spent viewing or age ratings. The trend of considering screen violence as a risk that is adjustable based on content characteristics and contextual factors has gained popularity in public health commentaries (The Lancet Regional Health–Americas, 2023). Experimental interventions also provide evidence that meticulously crafted media content can alter behavior regarding safety in the real world (Kjærvik and Bushman, 2023), supporting the importance of paying attention to how narratives are framed and messages are presented. This means that policymakers should focus on context-sensitive regulations and media literacy programs that focus on glamorization, justification, and reward systems around on-screen violence. For parents and those who care for them, the findings indicate active mediation interventions that focus on discussion and co-viewing where possible, and critically examining the motivation of the protagonist characters and the implications. The study of particular content types in early childhood also shows that specific types of on-screen content may be related to mental health indicators differently (Wang et al., 2024), explaining why the importance of content-sensitive advice cannot be overestimated.

There are a number of limitations that should be considered. The cross-sectional design did not allow strong causal inference or the ability to separate socialization and selection effects, such as the potentially important possibility that more violent youth selectively read and identify with narratives of violence. The longitudinal media research points to the need to distinguish between within-person and between-person mechanisms that would not confound the trait characteristics that stay constant with media-induced change (Lacko et al., 2024; Teng and Bushman, 2026). The use of self-report measures may have also introduced shared method variance and social desirability bias. In addition, behavioral or psychophysiological signs of desensitization were not studied, although the latter have been shown to be able to detect subtle changes in empathic responding to violent stimuli (Miedzobrodzka et al., 2023).

Future studies should be conducted using longitudinal and multi-method designs that can answer whether identification mediates the exposure-aggression relationship prospectively and whether reinforcing spirals are time-dependent. Particularly, the definition of prospective designs is relevant due to inconclusive results on the topic of violent media and empathy in both experimental and longitudinal studies (Lengersdorff et al., 2023; Ritchie et al., 2024). The distinction between the following forms of Malayalam violence representation, including morally approved vigilantism and aggression that is condemned, might help to understand when identification reinforces or neutralizes detrimental effects. The combination of objective measures of exposure and behavioral indicators would provide stronger support to inference and bring this cinema-specific literature into the wider practices of fine-tuning methodological principles in the study of violent media (Teng and Bushman, 2026).

## Data Availability

The original contributions presented in the study are included in the article/supplementary material, further inquiries can be directed to the corresponding author/s.
